# An Investigation of Two-Dimensional Ultrasound Carotid Plaque Presence and Intima Media Thickness in Middle-Aged South Asian and European Men Living in the United Kingdom

**DOI:** 10.1371/journal.pone.0123317

**Published:** 2015-04-17

**Authors:** Nazim Ghouri, David Purves, Kevin A. Deans, Greig Logan, Alex McConnachie, John Wilson, Jason M. R. Gill, Naveed Sattar

**Affiliations:** 1 BHF Glasgow Cardiovascular Research Centre, Institute of Cardiovascular and Medical Sciences, College of Medical, Veterinary and Life Sciences, University of Glasgow, Glasgow, G12 8TA, United Kingdom; 2 Robertson Centre for Biostatistics, Institute of Health and Wellbeing, University of Glasgow, Glasgow, G12 8QA, United Kingdom; 3 Department of Clinical Biochemistry, Aberdeen Royal Infirmary, Aberdeen, AB25 2ZN, United Kingdom; 4 Human Potential Centre, AUT University, Auckland, 1142, New Zealand; University of Oxford, UNITED KINGDOM

## Abstract

**Objectives:**

Ultrasound studies of carotid intima media thickness (cIMT) and plaques are limited in South Asians, a group at elevated cardiovascular disease (CVD) risk. We determined whether South Asians have a difference in these ultrasound markers compared to Europeans living in the United Kingdom and whether measured risk factor(s) could account for any such differences.

**Methods:**

One hundred South Asian men, aged 40 to 70 years and 100 European men of similar age and BMI, without diagnosed CVD or diabetes, underwent carotid ultrasound for measurement of cIMT and carotid plaque presence. Physical activity, cardiorespiratory fitness, anthropometry and blood pressure were assessed, fasted blood taken for measurement of cardiometabolic risk factors and demographic and lifestyle factors recorded.

**Results:**

Age-adjusted mean (SD) cIMT was similar in South Asians and Europeans (0.64 (0.16) mm v 0.65 (0.12) mm, p = 0.64). Plaque was present in 48 South Asians and 37 Europeans and overall, there was no age-adjusted difference between South Asian and Europeans for plaque score(odds ratio 1.49, 95% CI, 0.86-2.80, p = 0.16), however, South Asians appeared to have more plaques at a younger age than Europeans; at age 40-50 years the odds of South Asians having plaques was 2.63 (95% CI, 1.16-5.93) times that for Europeans.

**Conclusions:**

cIMT is similar between healthy South Asian and European men. Whilst there was no overall difference in plaque presence in South Asians, there is an indication of greater plaque prevalence at younger ages - an observation requiring further investigation. Prospective studies linking plaques to CVD outcomes in South Asians are needed to investigate whether these measures help improve CVD risk prediction.

## Introduction

South Asians living in Europe and North America have greater cardiovascular disease (CVD) risk than the background population of white European descent [1]. Several studies have shown that coronary heart disease (CHD) morbidity and mortality is higher, and manifests about 10 years earlier in life, in migrant South Asians than in white European populations [2–4]. Similarly, deaths from cerebrovascular disease are higher in migrant South Asians than Europeans [5]. The increased CHD burden in migrant South Asians is not fully explained by traditional CVD risk factors [6–8], Moreover, several current risk models do not account for the excess CVD risk in South Asians—e.g. SCORE—and thus identification of other markers to identify the increased CVD risk in South Asians is a clinical concern [9].

Carotid intima media thickness (cIMT) is recognised as a surrogate risk factor for CHD and stroke [10]. Key strengths of this method is that it is noninvasive, inexpensive and the availability of semi-automated software to read images and measure cIMT [11], making it a relatively quick, accurate and potentially reproducible screening modality. Currently there are no published prospective longitudinal data on cIMT and CVD outcomes on South Asians and there are only two cross-sectional studies comparing cIMT between South Asians and Europeans from the same population [12;13]. Data published by Chaturvedi et al suggested that cIMT was similar between both ethnic groups, with both groups containing similar numbers of subjects with known CVD [13]. Data from Anand and colleagues indicated that cIMT was in fact lowering South Asians compared with Europeans, despite South Asians having a higher number of CVD events [12], thus suggesting that absolute cIMT values may not be sensitive enough to capture or reflect the excess CVD risk in South Asians.

Two-dimensional assessment of carotid plaque has also been used as a modality to facilitate CVD risk stratification and is also associated with future risk of myocardial infarction and stroke [14–16]. Furthermore, when incorporated into risk stratification models, carotid plaque presence/absence enhances risk reclassification independently of cIMT [17;18].

Thus the aims of this study were to determine: (i) whether South Asians have different cIMT or carotid plaque presence compared to Europeans; and if so, (ii) whether differences in carotid plaque presence between South Asians and Europeans occur at an earlier age than differences in cIMT; and (iii) to determine whether any measured risk factors could account for any observed differences in cIMT and/or carotid plaque presence.

## Methods

### Study population

One hundred South Asian (defined as having both parents of Indian, Pakistani, Bangladeshi or Sri Lankan origin) and 100 European (both parents of white European origin) men living in the UK, aged 40–70, without known cardiometabolic disease (coronary heart disease, cerebrovascular disease, peripheral vascular disease, or diabetes) were recruited into the cross-sectional **C**arotid **U**ltrasound and **R**isk of **V**ascular disease in **E**uropeans and **S**outh Asians (CURVES) study. The study was approved by the West of Scotland Research Ethics Committee and conducted according to the principles expressed in the Declaration of Helsinki. All participants gave written informed consent. Full descriptive details of the study design, cohort (including method of recruitment) and recording of health history, including smoking status and family health history, education, socioeconomic status information and dietary information have been previously described in detail [19]. Publicity for recruitment made clear reference to eligibility criteria and only eligible potential participants in primary care were written to. Exclusion criteria were re-checked with interested participants directly when they were contacted to discuss the study in more detail and arranging screening visits,. It was not necessary to exclude any interested volunteers from participation. Of the 100 South Asian men recruited, 89 were of Pakistani origin, 10 were of Indian origin and 1 man was of Sri Lankan origin.

### Metabolic, anthropometric and physical assessment

Each participant underwent a comprehensive assessment of metabolic, body composition, cardiorespiratory fitness and physical activity profile as described previously [19]. Metabolic assessment included fasting venous blood analysis for glucose, HbA1c, total cholesterol, HDL cholesterol, triglyceride (TG), alanine aminotransferase (ALT) and insulin; as well as blood pressure measurement. Anthropometric measurements including height, body mass and waist circumference (midpoint between the lower costal margin and iliac crest) were measured in all participants by the same person, trained in undertaking anthropometric measurements in accordance with international standard protocols [20]. Total body lean and fat masses were measured using air displacement plethysmography. Cardiorespiratory fitness was determined through maximum oxygen uptake during an incremental treadmill test to exhaustion. Objectively measured physical activity was calculated from data obtained from accelerometers for up to 7 consecutive days [19].

### Carotid ultrasound scan protocol and analysis

All participants were scanned by NG, using the same machine (Siemens Acuson Sequoia 512 scanner) with an L8 5–12 MHz linear array broadband transducer (Siemens Medical Solutions, Erlangen, Germany). NG was trained by KAD to the American Society of Echocardiography (ASE) standards for image quality by ultrasound scanning [21]. The protocol for scanning created for the study was adapted from the pSoBid study scanning protocol, which measured and analysed the same carotid ultrasound outcomes of interest [22]. In summary, with the subject supine, the right carotid artery was scanned and then the left, with the head turned 45 degrees to the contralateral side. B mode still images and dynamic clips were recorded from three sites in the following order: distal 2cm of the common carotid artery, the carotid bulb, and the proximal internal carotid artery. Prior to obtaining the B-mode images and clips, Doppler analysis of each internal carotid artery was carried out in order to exclude pre-existing significant stenosis. If Doppler velocity was above 1.25m/s, indicative of pathological stenosis, the participant was excluded from the study and subsequent data analysis, and appropriate clinical advice was sought from the Stroke Team at the Western Infirmary, Glasgow regarding further management. No South Asians and only one European were excluded on this basis.

Scans were analysed for cIMT measurement and carotid plaque presence using validated semi-automated software (Siemens—Syngo US Workplace release 3.5), which included the Arterial Health Package (AHP) [23]. Anonymised images were saved as Digital Imaging and Communications in Medicine (DICOM) files and burned to CD to enable blinded offline analysis with all scans analysed after study recruitment had been completed. All scans were analysed for cIMT measurement by the same blinded reader (GL). Carotid plaque presence for all scans was determined by the same individual (KAD). A plaque was defined as a ‘focal structure encroaching into the arterial lumen of at least 0.5 mm or 50% of the surrounding IMT value, or demonstrating a thickness >1.5 mm as measured from the media-adventitia interface to the intima-lumen interface’ [24]. To adjust for any unreadable video clips, the plaque count was then converted into a plaque score by dividing this value by the number of readable images present and multiplying the outcome by six (the maximum possible number of images per participant) [14]. GL’s reproducibility for cIMT measurement fell within the ASE intra-reader reproducibility standards [21]—each subject’s mean absolute difference in cIMT was <0.055mm with a coefficient of variation <6%. cIMT was measured in the distal 1cm of the right and left common carotid arteries and the mean per subject common carotid artery IMT was calculated.

### Data analysis

The sample size for the CURVES study was based on a the *a priori* assumption that prevalence of carotid plaques would be 50% higher in South Asians than Europeans, based on previous UK mortality data showing ~50% greater CHD mortality in South Asian compared to European men—the standardised mortality ratio (SMR) in English and Welsh men was 97 (95% CI, 96, 97); for Scottish men was 104 (95% CI, 96, 97); for Indian men was 131 (95% CI, 126, 137); for Pakistani men was 162 (95% CI, 152, 172); and for Bangladeshi men was 175 (158, 193) [5]. We also estimated that 40% of European men aged 40–70 would have plaque [22]. Thus, we determined that 97 men per ethnic group would be needed for 80% power to detect differences in plaque presence between the South Asian and European groups at the 5% significance level.

Summary statistics are presented for all variables for both South Asians and Europeans. Continuous variables were compared between the ethnic groups by t-tests (or Wilcoxon rank sum test for non-normally distributed variables) and Fisher’s exact test for categorical variables.

Linear regression was used to model cIMT as the outcome and logistic regression for plaque score and for modelling the predictors of plaque presence. The odds ratios for a South Asian having carotid plaque compared to Europeans are presented, with 95% CI and p-value, when ethnicity was the only predictor in the model and when adjusted for participant age together with select demographic, lifestyle and CHD risk factor variables. A composite group of predictors was selected using backwards elimination on each subgroup, to select variables significant at the 10% level for the final model.

The statistical software package R for Windows v2.14 was used for all analysis [25]. Statistical significance was accepted at p < 0.05.

## Results

### Demographic, metabolic and dietary variables

Baseline data for all 200 volunteers are presented in Table 1. Mean ± SD reported energy intake (expressed per kg body mass) did not differ between the Europeans and South Asians (19.8±6.0 vs 18.7±6.4 kcal.kg^-1^.day^-1^, p = 0.21). There were also no differences in reported protein, carbohydrate or fat intakes between the two groups (data not shown), but reported alcohol intake was higher in the Europeans than South Asians (21.9±18.3 vs 1.1±5.6 g.day^-1^, p<0.0001).

**Table 1:** Demographic and metabolic variables for all South Asian and European men.

		South Asian (N = 100)	European (N = 100)	p-value
***Demographic and lifestyle variables***				
Age (years)		49.4 (7.2)	49.7 (6.8)	0.755
Body mass (kg)		82.0 (12.1)	85.7 (13.9)	0.049
Height (m)		1.74 (0.06)	1.78 (0.06)	<0.0001
BMI (kg.m^-2^)		27.1 (3.9)	26.9 (4.2)	0.692
Years in Education		15.6 (3.5)	14.4 (3.2)	0.010
SIMD Quintile	1	9 (9.0%)	7 (7.0%)	
	2	10 (10.0%)	14 (14.0%)	
	3	17 (17.0%)	19 (19.0%)	0.620
	4	26 (26.0%)	18 (18.0%)	
	5	38 (38.0%)	42 (42.0%)	
Smoking Status	never-smoker	81 (81.0%)	54 (54.0%)	
	ex-smoker	6 (6.0%)	36 (36.0%)	<0.0001
	current	13 (13.0%)	10 (10.0%)	
Alcohol Consumption^b^	none	93 (93.0%)	10 (10.1%)	
(units per week)	≤ 20	5 (5.0%)	70 (70.7%)	<0.0001
	≥ 21	2 (2.0%)	19 (19.2%)	
Parental Diabetes Status^b^	yes	54 (54.0%)	13 (13.1%)	<0.0001
Sibling Diabetes Status^b^	yes	17 (17.0%)	2 (2.0%)	0.0004
***Metabolic variables***				
Glucose (mmol/L)		5.4 (5.0, 5.8)	5.1 (4.8, 5.4)	0.0005^a^
HbA1c (%)^c^		5.7 (5.5, 6.1)	5.4 (5.2, 5.6)	<0.0001^a^
HbA1c (mmol.mol^-1^)^b^		39.0 (37.0, 43.0)	36.0 (33.0, 38.0)	<0.0001^a^
Insulin (^mcU^.l^-1^)^b^		14.9 (7.4)	9.2 (5.3)	<0.0001
HOMA_IR_^b^		3.3 (2.4, 4.6)	1.8 (1.1, 2.7)	<0.0001^a^
Total Cholesterol (mmol/L)		5.3 (0.9)	5.6 (1.0)	0.053
HDL-Cholesterol (mmol/L)		1.1 (1.0, 1.3)	1.3 (1.0, 1.3)	<0.0001^a^
LDL-cholesterol (mmol/L)		3.4 (0.9)	3.6 (0.8)	0.457
Triglycerides (mmol/L)		1.7 (1.1)	1.5 (0.9)	0.0423^a^
AST (U.l^-1^)		23.8 (7.6)	25.1 (8.5)	0.242
ALT (U.l^-1^)		27.0 (22.0, 36.2)	26.0 (20.0, 35.2)	0.758^a^
GGT (U.l^-1^)		32.5 (22.0, 47.0)	28.0 (20.8, 42.8)	0.261^a^
CRP (mg.l^-1^)^c^		1.8 (1.1, 3.8)	1.2 (0.6, 2.5)	0.0028^a^
Systolic blood pressure (mm Hg)		126.8 (15.0)	127.6 (12.8)	0.686
Diastolic blood pressure (mm Hg)		78.2 (8.9)	74.9 (7.2)	0.004

Values are mean (SD) for normally distributed variables with p values calculated by t-tests and median (IQR) for non-normally distributed variables with p-values calculated by Wilcoxon test^a^. Categorical variable p values are calculated by Fisher's Exact test. ^b,c^n = 99 for South Asians and Europeans respectively. SIMD—Scottish Index of Multiple Deprivation; HOMA-IR

### Body composition, fitness and physical activity variables

Body composition, fitness and physical activity variables for all 200 volunteers are presented in Table 2. Waist-hip-ratio was higher, lean mass was lower and fat mass and percentage body fat were higher in the South Asians. Total adiposity and central adiposity factors were higher and body size units lower in South Asians than Europeans. Cardiorespiratory fitness (as assessed by VO_2max_) was lower in South Asians than Europeans.

**Table 2:** Body composition, fitness, and physical activity variables for all South Asian and European men.

	South Asian (N = 100)	European (N = 100)	p-value
***Body composition variables***			
***(a) Body circumferences***			
Waist (cm)	97.8 (10.9)	95.7 (11.2)	0.183
Hips (cm)	99.4 (6.6)	100.5 (7.1)	0.240
Waist-to-hip ratio	0.98 (0.07)	0.95 (0.07)	0.0009
***(b) Fat and lean mass*** ^***a***^			
Lean mass (kg)	57.1 (6.2)	63.1 (7.2)	<0.0001
Fat mass (kg)	24.5 (9.0)	21.8 (9.7)	0.044
Percentage body fat	29.3 (7.4)	24.9 (7.4)	<0.0001
***Fitness variables***			
VO_2 max_ (l.min^-1^)^a^	2.52 (0.43)	3.33 (0.63)	<0.0001
VO_2 max_ (ml.kg^-1^.min^-1^)^a^	31.2 (5.8)	39.4 (7.8)	<0.0001
***Physical activity variables*** ^***c*,*b***^			
Sedentary (% of wear time)	66.3 (10.0)	64.3 (9.0)	0.185
Light physical activity (% of wear time)	30.6 (9.6)	30.8 (8.0)	0.901
Moderate-to-vigorous physical activity (% of wear time)	2.8 (1.6, 4.2)	4.1 (3.0, 6.8)	<0.0001^a^
Total accelerometer wear time (min.day^-1^)	813.4 (90.6)	864.0 (74.4)	0.0001

Values are mean (SD) for normally distributed variables with p-values calculated by t-tests and median (IQR) for non-normally distributed variables with p-values calculated by Wilcoxon test^a^. Categorical variable p-values are calculated by Fisher's Exact test. ^a^n = 99 and ^c^n = 84 for Europeans;^b^n = 85 for South Asians.

South Asians also engaged in less moderate and vigorous physical activity than Europeans: the proportion of time spent in moderate-to-vigorous physical activity was ~29% lower in South Asians than Europeans. This equated to South Asians engaging in ~22 minutes of moderate-to-vigorous physical activity (MVPA) per day compared to ~31 minutes per day for the Europeans.

### Carotid ultrasound analysis

**Fig 1:**
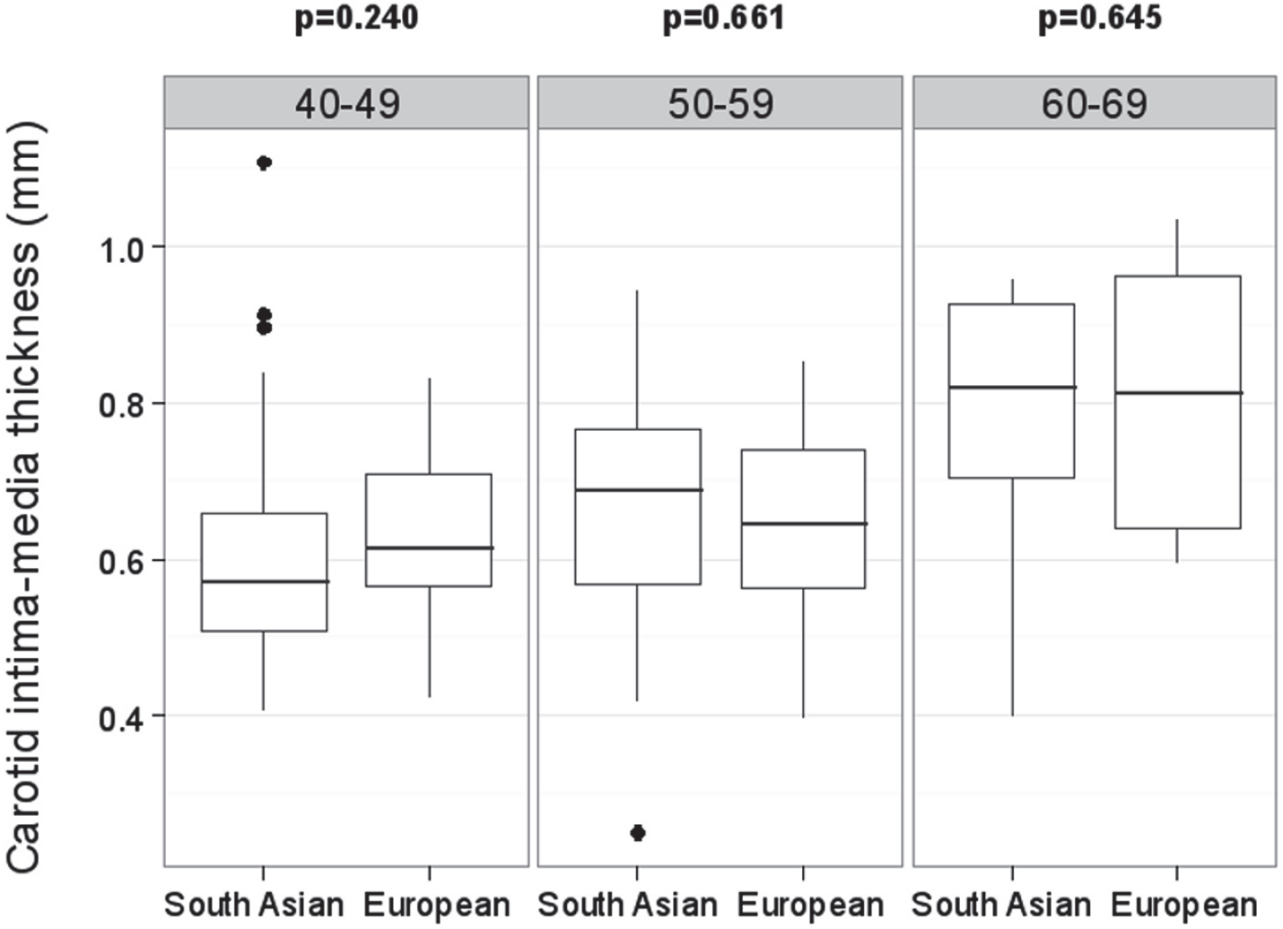
Carotid intima media thickness by age decade and ethnicity. Number of participants in each age decade (South Asian, European): 40–49 (55, 55); 50–59 (34, 36); 60–69 (11, 9)

Differences in ultrasound markers of atherosclerosis are presented in Table 3. There were no significant differences in unadjusted or age-adjusted mean cIMT between South Asians and Europeans. Fig 1 shows the differences in cIMT for each ethnic group, by age category. The expected trend of increasing mean cIMT with age was present for each ethnic group; however, there were no differences in mean cIMT between the ethnic groups in any age group. In regression analysis, cIMT was higher by 0.08 mm (95% CI, 0.06–0.11; p<0.001) for every ten year higher age, with a similar increase observed for each ethnic group (p = 0.177 for ethnicity by age interaction).

**Table 3:** Differences in cIMT and plaque scores between South Asian and European men.

	South Asian N = 100	European N = 100	South Asian vs. European	
			Estimate (95% CI)	p-value
***cIMT analysis***				
Mean (SD)	0.64 (0.16)	0.65 (0.12)	Difference^(^ ^a^ ^)^: -0.01 (-0.05, 0.03)	0.638
***Plaque Analysis***				
0 plaques	52 (52.0%)	63 (63.0%)		
1 to 2 plaques	38 (38.0%)	29 (29.0%)	Odds Ratio^(^ ^b^ ^)^: 1.49 (0.86, 2.60)	0.155
>2 plaques	10 (10.0%)	8 (8.0%)		

^(a)^linear regression, adjusted for age

^(b)^proportional odds regression, adjusted for age

Both systolic and diastolic blood pressure were associated with cIMT univariately and after adjustment for age, BMI and antihypertensive treatment (in adjusted analyses, cIMT increased by 0.03 mm (95% CI, 0.02–0.05; p<0.001) per 10 mmHg increase in systolic blood pressure and by 0.05 mm (95% CI,0.03–0.08; p<0.001) per 10 mmHg increase in diastolic blood pressure),and there were similar associations for each ethnic group.

### Ethnicity and plaque presence

As a result of inadequate image quality, three participants did not have a full complement of six video clips for the derivation of plaque score (two South Asians, with five readable clips each; one European, with two readable clips). There was no statistically significant evidence of increased carotid plaque score in South Asian men compared to European men (1.49, 95% CI, 0.86–2.80, p = 0.16) (Table 3). This remained the case when using plaque presence as the outcome—the odds ratio of plaque presence between South Asian and European men at (approximate sample mean) age 50 was 1.88, 95% CI, 0.83–4.27; p = 0.13 (S1 Table)). Even after we examined mediating or moderating effects of other risk factors on this difference, there remained no difference in plaque presence between the two groups.

**Fig 2:**
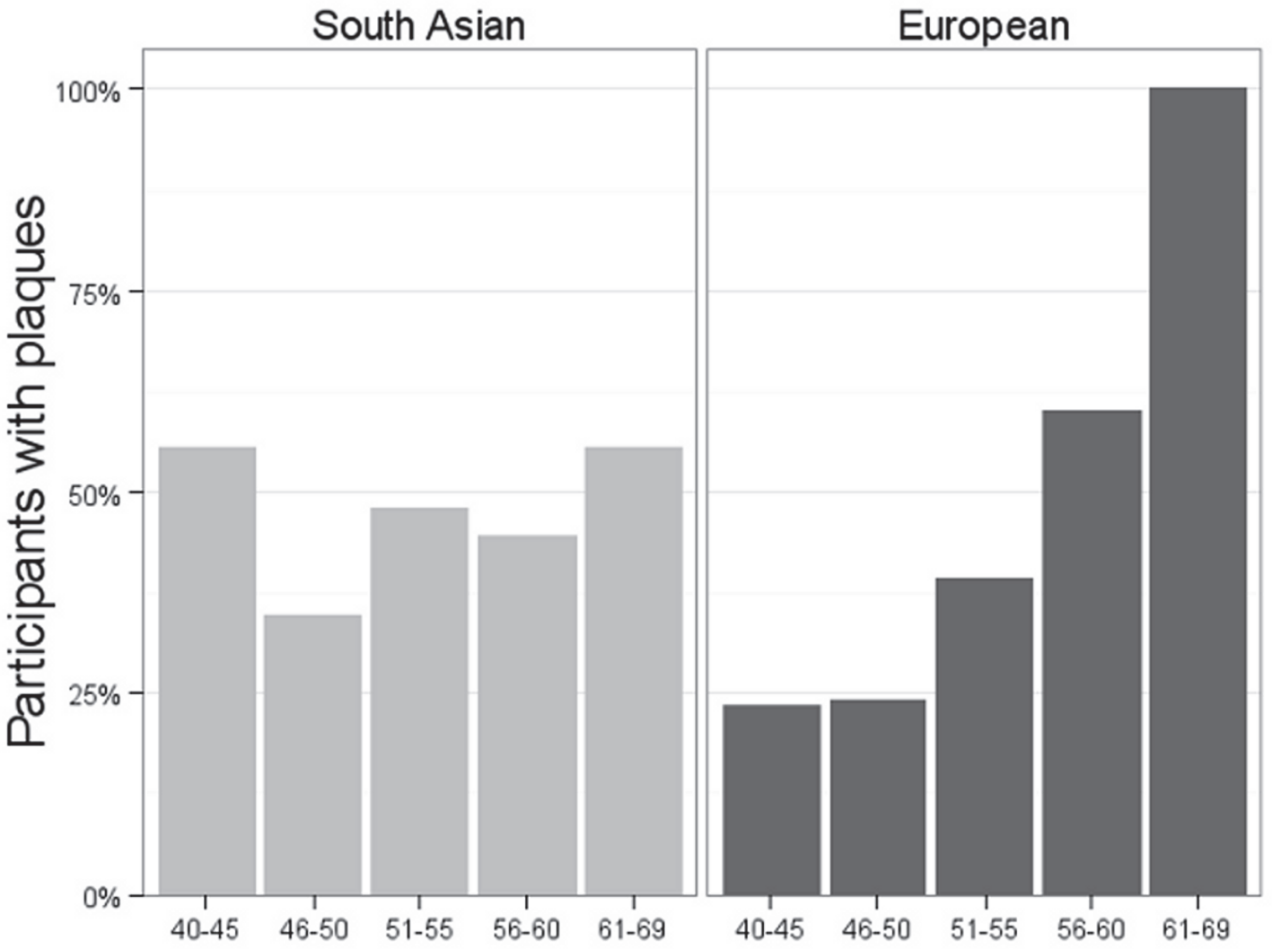
Presence of carotid plaque by ethnicity and age*. *Percentage of participants within each category. Number of participants in each age category (South Asian, European): 40–45 (36, 30); 46–50 (23, 29); 51–55 (23,23); 56–60 (9, 10); 61–69 (9, 8)

Of interest, plaques were more prevalent in the younger South Asians, as seen in Fig 2; in 40–50 year-olds, South Asians had 2.63 (95% CI, 1.16–5.93; p = 0.019) times the odds of plaques compared to Europeans, whereas there was no evidence of a difference at older age. Further, there was a significant interaction between ethnicity and age when predicting the odds of carotid plaques; the odds of plaques increased with age for European men, while no association with age was observed in South Asian men

## Discussion

The main finding from this study confirmed that there was no difference in cIMT despite there being the expected differences in the usual cardiometabolic risk factors observed in middle-aged South Asian compared to European men living in the UK. The other finding from this study was that there was no significant difference in carotid plaque presence, though pre-defined subgroup analysis suggested South Asians may have more plaques at younger ages with apparently no obvious increase in prevalence in older South Asians. However, it is possible that our exclusion criteria, which excluded participants with known vascular disease or diabetes, may have biased the study against observation of an ethnic difference in carotid plaques, particularly in the older subgroup, as prevalence of diabetes and premature cardiovascular disease is known to be higher in South Asians than Europeans [2;26;27].

The finding that cIMT is not increased in South Asians is consistent with the two previous published studies comparing South Asian men with those of European ancestry [12]. However unlike data presented in this analysis, the earlier data combines healthy subjects with those who have CVD. Thus the earlier data do not allow for conclusions to be made or hypothesised on the role of cIMT as a useful screening test in South Asians. Furthermore, novel published data on cIMT measurements in children living in the UK showed that cIMT levels in South Asian children were similar to those of European children [28]. These data in combination with this present analysis indicate that cIMT between South Asians and Europeans remains similar throughout life and hence may suggest that absolute cIMT values are not a sensitive marker for CVD risk in South Asians. Of interest, South Asians have narrower carotid artery diameter compared to Europeans, thus relative to vessel diameter, South Asians have a greater cIMT compared to Europeans [29] but the relevance of this finding is uncertain.

One possible explanation for the lack of difference in cIMT is that whilst atherosclerosis is chiefly an intimal process, IMT is approximately 80% media, and only 20% intima. Therefore any measurements made, and any changes in cIMT are likely to be a reflection of changes in the media [30] thus a key determinant of cIMT is the effect of blood pressure which is known to cause medial wall hypertrophy [31]. Data from this present analysis show that South Asians have similar systolic blood pressure compared to Europeans and whilst South Asians have higher diastolic blood pressure, the mean values fall well within the normal range for healthy adults. Further, a systematic review of blood pressure in South Asians showed no significant difference in blood pressure between South Asians and Europeans [32].

The aim of this study was to investigate plaque presence/quantity and not carotid plaque morphology. The reasons for this were two-fold. Firstly, one cross-sectional study which compared age-related cIMT differences with differences in carotid plaque disease between population groups of two socioeconomic extremes, demonstrated that differences between affluent and deprived populations in plaque score were apparent earlier in the life-course (~10 years) than differences in cIMT even after adjustment for a range of traditional and novel risk factors [22]—suggesting that plaque score may be a more useful marker of deprivation-associated CVD risk than cIMT. Thus, these intriguing data suggested the possibility that plaque score might be a more sensitive measure of CVD risk than cIMT and such data in South Asians was lacking. Secondly, atherosclerotic plaque morphology/size has been indicated to be different between South Asians and Europeans, with South Asians having more aggressive morphology in coronary arteries for example [33;34]. Thus the overall negative finding in this analysis should not be interpreted to indicate that South Asians are not more susceptible to atherosclerotic plaque or subsequent pathological manifestations from them. As noted above, we may have biased the results towards not seeing plaque differences in older age by virtue of our inclusions criteria required no CVD or known diabetes in participants.

This study has a number of specific strengths. To the authors’ knowledge, it is the first study measuring and comparing carotid plaque between South Asian with European adults in the UK. The groups were similar in age, BMI and socioeconomic status, and although the South Asians spent more years in education, smoked less and drank less alcohol than the Europeans, these factors were found not to significantly confound the key study outcomes. Furthermore, compared to the previous published data from Canada (which had a study population spanning a similar age-range and included individuals with and without known CVD), participants in this analysis were better matched for age and present clearer comparative data on individuals without CVD [12].

However, this study does have limitations. A degree of caution is warranted in extrapolating the findings to the general UK South Asian population or to women. By nature of the recruitment methods, the study cohort comprises self-selected groups of South Asians and Europeans, who may not be fully representative of the background populations. **We made a deliberate choice to exclude participants with known** vascular disease or diabetes as we were primarily concerned with evaluating differences in carotid atherosclerosis in the apparently healthy ‘at risk’ population, which would be confounded by inclusion of participants with existing disease. Our population included 13 South Asians (vs one European) with undiagnosed diabetes, who were not excluded from the data analysis. This proportion of undiagnosed diabetes in the present South Asian sample is similar to that observed in other Scottish South Asian [35] cohorts. In addition, the findings with respect to ethnic cIMT differences, fitness, physical activity, adiposity and metabolic variables in South Asians in the present study are consistent with the body of previously published data in this area [12;13;36–38], suggesting that our sample is likely to be reasonably representative of the ‘at risk’ South Asian population without diagnosed cardiometabolic disease. **However, as cardiometabolic disease** is more prevalent in South Asians than Europeans [2;27], with this ethnic difference increasing with age, our sample of South Asians may have been healthier than a representative overall population sample. This could potentially explain the lack of difference between groups in the vascular markers measured, particularly in the older subgroup. **A** more pragmatic approach if the study were to be repeated, would be to relax the inclusion criteria to include participants with known diabetes, given the premature onset and increased prevalence of diabetes of South Asians [26;27;39]. We recognise that the findings from this study cannot be extended to women. It was beyond the scope of this study to recruit women, however we acknowledge that novel data from the USA suggest that migrant South Asian women are also at increased risk of CVD relative to their European peers [40], thus future study in this group is warranted.

An important limitation of cIMT measurement and 2 dimensional plaque scoring is that change over time is limited because plaque propagates longitudinally along the artery wall ~2.4 times quicker than it extends into the arterial lumen [41] and data suggest that South Asians may have longer atherosclerotic lesions [33;34]. Validated three-dimensional (3D) ultrasound techniques exist and are demonstrated to possibly be more informative than existing 2D cIMT and plaque measures [42], which can detect earlier responses to intervention e.g. statin therapy over a six-month period [43]. However, published long-term longitudinal data or indeed CVD outcome data based on 3D imaging of carotid arteries is lacking. Finally, the cross-sectional nature of the study, with simultaneous assessment of exposure and outcome variables, means that conclusions on causality cannot be definitively made or refuted.

In conclusion, this present study strongly reaffirms that cIMT is similar between South Asian and European men despite greater risk factors in the former group. They also suggest no overall difference in plaques in South Asians although there is a strong suggestion for greater plaques at younger ages, an observation which requires further investigation and extension to women. Of course, ideally, prospective studies linking plaques to outcomes in South Asians are needed to investigate whether these measures help explain higher CVD risk in this high risk population.

## Supporting Information

S1 Table Adjusted odds ratios for plaque presence between South Asians and Europeans. (DOCX)
