# Supplementary material for: An Investigation of Two-Dimensional Ultrasound Carotid Plaque Presence and Intima Media Thickness in Middle-Aged South Asian and European Men Living in the United Kingdom
Source: PLoS One. 2015 Apr 17;10(4):e0123317. doi: 10.1371/journal.pone.0123317 (PMC4401566; doi:10.1371/journal.pone.0123317)
Supplement: S1 Table — (DOCX) [file pone.0123317.s001.docx]

**S1 Table: Adjusted odds ratios for plaque presence between South Asians and Europeans**

|  | **Odds Ratio(95% CI)** | **p-value** |
| --- | --- | --- |
| Unadjusted | 1.57 (0.89, 2.77) | 0.116 |
| Age^+^ (quadratic) at 50 years | 1.88 (0.83, 4.27) | 0.130 |
| BMI | 1.89 (0.83, 4.30) | 0.129 |
|  |  |  |
|  |  |  |
|  |  |  |
| Combined adiposity measures^d^ | 2.09 (0.82, 5.37) | 0.122 |
|  |  |  |
|  |  |  |
| Traditional risk factors^b^ | 1.90 (0.76, 4.73) | 0.167 |
|  | 1.67 (0.70, 3.96) | 0.246 |
|  | 1.77 (0.69, 4.54) | 0.232 |
|  | 1.83 (0.71, 4.71) | 0.208 |
| Classic risk factors + insulin resistance + glucose | 1.76 (0.68, 4.56) | 0.243 |
| Metabolic risk factors^c^ | 1.60 (0.67, 3.80) | 0.288 |
| Novel risk factors^d^ | 1.89 (0.82, 4.35) | 0.131 |
| Moderate to vigorous physical activity | 1.89 (0.75, 4.76) | 0.176 |
| VO_2max_* | 1.83 (0.74, 4.56) | 0.190 |
| Final Model^h^** | 1.30 (0.67, 2.53) | 0.097 |

Table shows the relative effect of ethnicity, adjusted for age (quadratic) and their interaction, on the odds of plaques and further adjusted by the stated variables.
^a^Combined adiposity measures derived using factor analysis of available measures, leading to 3 composite measures of adiposity: of skinfold measures (suprailiac, supraspinale, triceps, subscapular, thigh, calf, biceps and fat mass), body size (lean mass, height, mid-thigh, mid-calf and hip circumference) and central fats (waist circumference and waist-to-hip ratio).

^b^Traditional risk factors: Total cholesterol, HDL cholesterol, smoking status, systolic blood pressure and diastolic blood pressure
^c^Metabolic risk factors: HDL cholesterol, triglycerides and insulin

^d^Novel risk factors: CRP, AST, ALT and GGT
^e^Final model: Ethnicity and age (quadratic) interaction and HDL.
^+^: at age 50 years
* ml.kg^-1^.min^-1^
** Significant predictors selected from variable subgroups by backward selection.
